# A generative model of memory construction and consolidation

**DOI:** 10.1038/s41562-023-01799-z

**Published:** 2024-01-19

**Authors:** Eleanor Spens, Neil Burgess

**Affiliations:** 1 UCL Institute of Cognitive Neuroscience, University College London, London, UK; 2 UCL Queen Square Institute of Neurology, University College London, London, UK

**Keywords:** Computational neuroscience, Cognitive neuroscience

## Abstract

Episodic memories are (re)constructed, share neural substrates with imagination, combine unique features with schema-based predictions and show schema-based distortions that increase with consolidation. Here we present a computational model in which hippocampal replay (from an autoassociative network) trains generative models (variational autoencoders) to (re)create sensory experiences from latent variable representations in entorhinal, medial prefrontal and anterolateral temporal cortices via the hippocampal formation. Simulations show effects of memory age and hippocampal lesions in agreement with previous models, but also provide mechanisms for semantic memory, imagination, episodic future thinking, relational inference and schema-based distortions including boundary extension. The model explains how unique sensory and predictable conceptual elements of memories are stored and reconstructed by efficiently combining both hippocampal and neocortical systems, optimizing the use of limited hippocampal storage for new and unusual information. Overall, we believe hippocampal replay training generative models provides a comprehensive account of memory construction, imagination and consolidation.

## Abstract (web summary)

Spens and Burgess develop a computational model that shows how the hippocampus encodes episodic memories and replays them to train generative models of the world. Conceptual and sensory representations of experience can then be recombined for imagination and memory.

## Main

Episodic memory concerns autobiographical experiences in their spatiotemporal context, whereas semantic memory concerns factual knowledge^1^. The former is thought to rapidly capture multimodal experience via long-term potentiation in the hippocampus, enabling the latter to learn statistical regularities over multiple experiences in the neocortex^2–5^. Crucially, episodic memory is thought to be constructive; recall is the (re)construction of a past experience, rather than the retrieval of a copy^6,7^. But the mechanisms behind episodic (re)construction and its link to semantic memory are not well understood.

Old memories can be preserved after hippocampal damage despite amnesia for recent ones^8^, suggesting that memories initially encoded in the hippocampus end up being stored in neocortical areas, an idea known as ‘systems consolidation’^9^. The standard model of systems consolidation involves transfer of information from the hippocampus to the neocortex^2–4,10^, whereas other views suggest that episodic and semantic information from the same events can exist in parallel^11^. Hippocampal ‘replay’ of patterns of neural activity during rest^12,13^ is thought to play a role in consolidation^14,15^. However, consolidation does not just change which brain regions support memory traces; it also converts them into a more abstract representation, a process sometimes referred to as semanticization^16,17^.

Generative models capture the probability distributions underlying data, enabling the generation of realistic new items by sampling from these distributions. Here we propose that consolidated memory takes the form of a generative network, trained to capture the statistical structure of stored events by learning to reproduce them (see also refs. ^18,19^). As consolidation proceeds, the generative network supports both the recall of ‘facts’ (semantic memory) and the reconstruction of experience from these ‘facts’ (episodic memory), in conjunction with additional information from the hippocampus that becomes less necessary as training progresses.

This builds on existing models of spatial cognition in which recall and imagination of scenes involve the same neural circuits^20–22^, and is supported by evidence from neuropsychology that damage to the hippocampal formation (HF) leads to deficits in imagination^23^, episodic future thinking^24^, dreaming^25^ and daydreaming^26^, as well as by neuroimaging evidence that recall and imagination involve similar neural processes^27,28^.

We model consolidation as the training of a generative model by an initial autoassociative encoding of memory through ‘teacher–student learning’^29^ during hippocampal replay (see also ref. ^30^). Recall after consolidation has occurred is a generative process mediated by schemas representing common structure across events, as are other forms of scene construction or imagination. Our model builds on: (1) research into the relationship between generative models and consolidation^18,19^, (2) the use of variational autoencoders to model the hippocampal formation^31–33^ and (3) the view that abstract allocentric latent variables are learned from egocentric sensory representations in spatial cognition^22^.

More generally, we build on the idea that the memory system learns schemas which encode ‘priors’ for the reconstruction of input patterns^34,35^. Unpredictable aspects of experience need to be stored in detail for further learning, while fully predicted aspects do not, consistent with the idea that memory helps to predict the future^36–39^. We suggest that familiar components are encoded in the autoassociative network as concepts (relying on the generative network for reconstruction), while novel components are encoded in greater sensory detail. This is efficient in terms of memory storage^40–42^ and reflects the fact that consolidation can be a gradual transition, during which the autoassociative network supports aspects of memory not yet captured by the generative network. In other words, the generative network can reconstruct predictable aspects of an event from the outset on the basis of existing schemas, but as consolidation progresses, the network updates its schemas to reconstruct the event more accurately until the formerly unpredictable details stored in HF are no longer required.

Our model draws together existing ideas in machine learning to suggest an explanation for the following key features of memory, only subsets of which are captured by previous models:

1. The initial encoding of memory requires only a single exposure to the event and depends on the HF, while the consolidated form of memory is acquired more gradually^2,3,10^, as in the complementary learning systems (CLS) model^4^.
2. The semantic content of memories becomes independent of the HF over time^43–45^, consistent with CLS.
3. Vivid, detailed episodic memory remains dependent on HF^46^, consistent with multiple trace theory^11^ (but not with CLS).
4. Similar neural circuits are involved in recall, imagination and episodic future thinking^27,28^, suggesting a common mechanism for event generation, as modelled in spatial cognition^22^.
5. Consolidation extracts statistical regularities from episodic memories to inform behaviour^47,48^, and supports relational inference and generalization^49^. The Tolman–Eichenbaum machine (TEM)^31^ simulates this in the domain of multiple tasks with common transition structures (see also ref. ^50^), while ref. ^51^ models how both individual examples and statistical regularities could be learned within HF.
6. Post-consolidation episodic memories are more prone to schema-based distortions in which semantic or contextual knowledge influences recall^6,52^, consistent with the behaviour of generative models^32^.
7. Neural representations in the entorhinal cortex (EC) such as grid cells^53^ are thought to encode latent structures underlying experiences^31,54^, and other regions of the association cortex, such as the medial prefrontal cortex (mPFC), may compress stimuli to a minimal representation^55^.
8. Novelty is thought to promote encoding within HF^56^, while more predictable events consistent with existing schemas are consolidated more rapidly^57^. Activity in the hippocampus can reflect prediction error or mismatch novelty^58,59^, and novelty is thought to affect the degree of compression of representations in memory^60^ to make efficient use of limited HF capacity^42^.
9. Memory traces in the hippocampus appear to involve a mixture of sensory and conceptual features, with the latter encoded by concept cells^61^, potentially bound together by episode-specific neurons^62^. Few models explore how this could happen.

### Consolidation as the training of a generative model

**Fig. 1:**
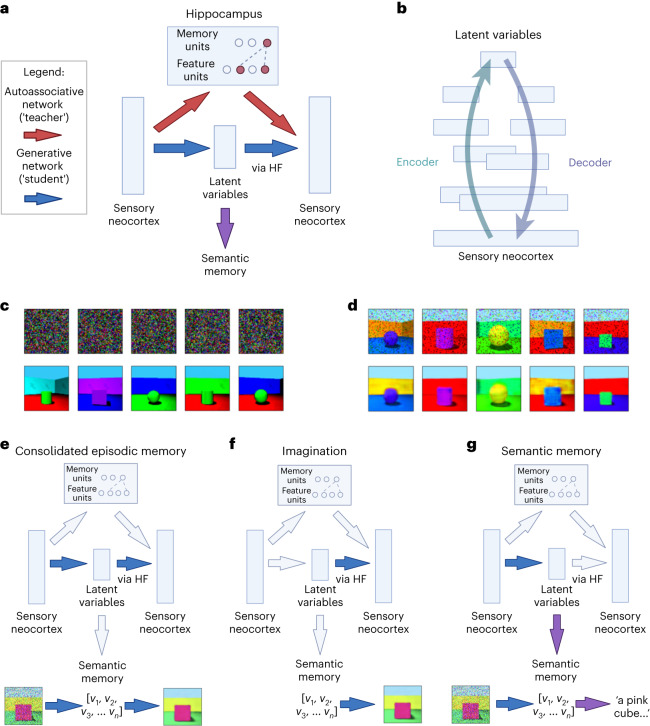
Architecture of the basic model. **a**, First, the hippocampus rapidly encodes an event, modelled as one-shot memorization in an autoassociative network (an MHN). Then, generative networks are trained on replayed representations from the autoassociative network, learning to reconstruct memories by capturing the statistical structure of experienced events. **b**, A more detailed schematic of the generative network to indicate the multiple layers of, and overlap between, the encoder and decoder (where layers closer to the sensory neocortex overlap more). The generation of a sensory experience, for example, visual imagery, requires the decoder to the sensory neocortex via HF. **c**, Random noise inputs to the MHN (top row) reactivate its memories (bottom row) after 10,000 items from the Shapes3D dataset are encoded, with five examples shown. **d**, The generative model (a variational autoencoder) can recall images (bottom row) from a partial input (top row), following training on 10,000 replayed memories sampled from the MHN. **e**, Episodic memory after consolidation: a partial input is mapped to latent variables whose return projections to the sensory neocortex via HF then decode these back into a sensory experience. **f**, Imagination: latent variables are decoded into an experience via HF and return projections to the neocortex. **g**, Semantic memory: a partial input is mapped to latent variables, which capture the ‘key facts’ of the scene. The bottom rows of **e**–**g** illustrate these functions in a model that has encoded the Shapes3D dataset into latent variables (*v*_1_, *v*_2_, *v*_3_, …, *v*_*n*_). Diagrams were created using BioRender.com.

Our model simulates how the initial representation of memories can be used to train a generative network, which learns to reconstruct memories by capturing the statistical structure of experienced events (or ‘schemas’). First, the hippocampus rapidly encodes an event; then, generative networks gradually take over after being trained on replayed representations from the hippocampus. This makes the memory more abstracted, more supportive of generalization and relational inference, but also more prone to gist-based distortion. The generative networks can be used to reconstruct (for memory) or construct (for imagination) sensory experience, or to support semantic memory and relational inference directly from their latent variable representations (see Fig. 1).

Before consolidation, the hippocampal autoassociative network encodes the memory. A modern Hopfield network (MHN)^63^ is used, which can be interpreted such that the feature units activated by an event are bound together by a memory unit^64^ (see Methods and Supplementary Information). Teacher–student learning^29^ allows transfer of memories from one neural network to another during consolidation^30^. Accordingly, we use outputs from the autoassociative network to train the generative network: random inputs to the hippocampus result in the reactivation of memories, and this reactivation results in consolidation. After consolidation, generative networks encode the information contained in memories. Reliance on the generative networks increases over time as they learn to reconstruct a particular event.

Specifically, the generative networks are implemented as variational autoencoders (VAEs), which are autoencoders with special properties such that the most compressed layer represents a set of latent variables, which can be sampled from to generate realistic new examples corresponding to the training dataset^65,66^. Latent variables can be thought of as hidden factors behind the observed data, and directions in the latent space can correspond to meaningful transformations (see Methods). The VAE’s encoder ‘encodes’ sensory experience as latent variables, while its decoder ‘decodes’ latent variables back to sensory experience. In psychological terms, after training on a class of stimuli, VAEs can reconstruct such stimuli from a partial input according to the schema for that class, and generate novel stimuli consistent with the schema. (Our use of VAEs is illustrative, and we would expect a range of other generative latent variable models, such as predictive coding networks^67–69^, to show similar behaviour.) See Methods and Supplementary Information for further details.

Generative networks capture the probability distributions underlying events, or ‘schemas’. In other words, here ‘schemas’ are rules or priors (expected probability distributions) for reconstructing a certain type of stimulus (for example, the schema for an office predicts the presence of co-occurring objects such as desks and chairs, facilitating episode generation), whereas concepts represent categories but not necessarily how to reconstruct them. However, schemas and concepts are closely related, and their meanings can overlap, with conflicting definitions in the psychology literature^70,71^.

During perception, the generative model provides an ongoing estimate of novelty from its reconstruction error (also known as ‘prediction error’, the difference between input and output representations). Aspects of an event that are consistent with previous experience (that is, with low reconstruction error) do not need to be encoded in detail in the autoassociative ‘teacher’ network^36–39^. Once the generative network’s reconstruction error is sufficiently low, the hippocampal trace is unnecessary, freeing up capacity for new encodings. However, we have not simulated decay, deletion or capacity constraints in the autoassociative memory part of the model.

### Combining conceptual and sensory features in episodic memory

Consolidation is often considered in terms of fine-grained sensory representations updating coarse-grained conceptual representations, for example, the sight of a particular dog updating the concept of a dog. Modelling hippocampal representations as sensory-like is a reasonable simplification, which we make in simulations of the ‘basic’ model in Fig. 1. However, memories probably bind together representations along a spectrum from coarse-grained and conceptual to fine-grained and sensory. For example, the hippocampal encoding of a day at the beach is likely to bind together coarse-grained concepts such as ‘beach’ and ‘sea’ along with sensory representations such as the melody of an unfamiliar song or the sight of a particular sandcastle, consistent with the evidence for concept cells in the hippocampus^61^. (This also fits with the observation that ambiguous images ‘flip’ between interpretations in perception but are stable when held in memory^72^, reflecting how the conceptual content of memories constrains recall.)

Furthermore, encoding every sensory detail in the hippocampus would be inefficient (elements already predicted by conceptual representations being redundant); an efficient system should take advantage of shared structure across memories to encode only what is necessary^40,41^. Accordingly, we suggest that predictable elements are encoded as conceptual features linked to the generative latent variable representation, while unpredictable elements are encoded in a more detailed and veridical form as sensory features.

**Fig. 2:**
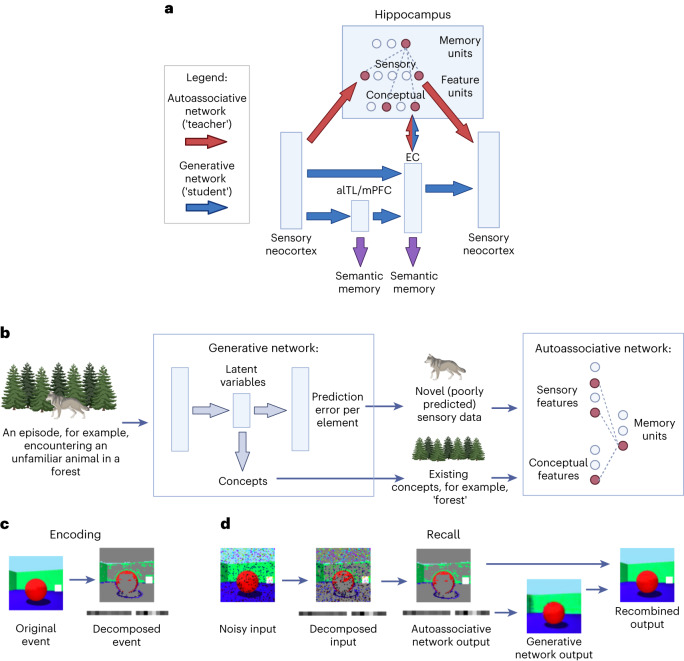
Architecture of the extended model. **a**, Each scene is initially encoded as a combination of predictable conceptual features related to the latent variables of the generative network and unpredictable sensory features that were poorly predicted by the generative network. An MHN (in red) encodes both sensory and conceptual features (with connections to the sensory neocortex and latent variables in EC, respectively), binding them together via memory units. Memories may eventually be learned by the generative model (in blue), but consolidation can be a prolonged process, during which time the generative network provides schemas for reconstruction and the autoassociative network supports new or detailed information not yet captured by these schemas. Multiple generative networks can be trained concurrently, with different networks optimized for different tasks. This includes networks with latent variables in EC, mPFC and alTL, each with its own semantic projections. However, in all cases, return projections to the sensory neocortex are via HF. **b**, An illustration of encoding in the extended model. **c**, Encoding ‘scenes’ from the Shapes3D dataset, with each ‘scene’ decomposed into unpredicted sensory features (top) and conceptual features linked to the generative network’s latent variables (bottom). Novel features (white squares overlaid on the image with varying opacity) are added to each ‘scene’. **d**, Recalling ‘scenes’ (with novel features) from the Shapes3D dataset. First, the input is decomposed; then, the MHN performs pattern completion on both sensory and conceptual features. The conceptual features (which in these simulations are simply the generative network’s latent variables) are then decoded into a schema-based prediction, onto which any stored sensory features are overwritten. Diagrams were created using BioRender.com.

Suppose someone sees an unfamiliar animal in the forest (Fig. 2b). Much of the event might be consistent with an existing forest schema, but the unfamiliar animal would be novel. In the extended model (Fig. 2 and section ‘Combining conceptual and unpredictable sensory features’), the reconstruction error per element of the experience is calculated by the generative model during perception, and elements with high reconstruction error are encoded in the autoassociative network as sensory features, along with conceptual features linked to the generative model’s latent variable representation. In other words, each pattern is split into a predictable component (approximating the generative network’s prediction for the pattern), plus an unpredictable component (elements with high prediction error). This produces a sparser vector than storing every element in detail, increasing the capacity of the network^42^.

### Neural substrates of the model

Which brain regions do the components of this model represent? The autoassociative network involves the hippocampus binding together the constituents of a memory in the neocortex, whereas the generative network involves neocortical inputs projecting to latent variable representations in the higher association cortex, which then project back to the neocortex via the HF. The entorhinal (EC), medial prefrontal cortex (mPFC) and anterolateral temporal lobe (alTL) are all prime candidates for the site of latent variable representations.

First, the EC is the main route between the hippocampus and the neocortex, and is where grid cells, which are thought to be a latent variable representation of spatial or relational structure^31,54^, are most often observed^73^. Second, mPFC and its connections to HF play a crucial role in episodic memory processing^70,74–78^, are thought to encode schemas^57,71^, are implicated in transitive inference^79^ and the integration of memories^80^, and perform dimensionality reduction by compressing irrelevant features^55^. Third, the anterior and lateral temporal cortices associated with semantic memory^81^ and retrograde amnesia^82^ probably contain latent variable representations capturing semantic structure. This might correspond to the ‘anterior temporal network’ associated with semantic dementia^83^, while the first network (between sensory and entorhinal cortices) might correspond to the ‘posterior medial network’^83^, and to the network mapping between visual scenes and allocentric spatial representations^20–22^.

Which regions constitute the generative network’s decoder? The decoder converts latent variable representations in the higher association cortex back to sensory neocortical representations via HF. Patients with damage to the hippocampus proper but not the EC can generate simple scenes (or fragments thereof), but an intact hippocampus is required for more coherent imagery of complex ones^23^. We hypothesize that conceptual units in the hippocampus proper help to generate complex, conceptually coherent scenes (perhaps through a recurrent ‘clean up’ mechanism), but that an intact EC and its return pathway to the sensory neocortex (the ventral visual stream for images) can still decode representations to some extent in their absence.

Multiple generative networks can be trained concurrently from a single autoassociative network through consolidation, with different networks optimized for different tasks. In other words, multiple networks could update their parameters to minimize prediction error on the basis of the same replayed memories. This could consist of a primary VAE with latent variables in the EC, plus additional parallel pathways from the higher sensory cortex to the EC via latent variables in the mPFC or the alTL. (Computationally, the shared connections could be fixed as the alternative pathways are trained.) Note that in all cases, return projections to the sensory neocortex via HF are required to decode latent variables into sensory experiences.

## Results

### Modelling encoding and recall

Each new event is encoded as an autoassociative trace in the hippocampus, modelled as an MHN. Two properties of this network are particularly important: memorization occurs with only one exposure, and random inputs to the network retrieve stored memories sampled from the whole set of memories (modelling replay).

We model recall as (re)constructing a scene from a partial input. First, we simulate encoding and replay in the autoassociative network. The network memorizes a set of scenes, representing events, as described above. When the network is given a partial input, it retrieves the closest stored memory. Even when the network is given random noise, it retrieves stored memories (see Fig. 1c). Second, we simulate recall in the generative network trained on reactivated memories from the autoassociative network, which is able to reconstruct the original image when presented with a partial version of an item from the training data (Fig. 1d).

In the basic model (Fig. 1a), the prediction error could be calculated for each event so that only the unpredictable events are stored in the hippocampus, as the predictable ones can already be retrieved by the generative network (however, this is not simulated explicitly). In the extended model (Fig. 2 and section ‘Combining conceptual and unpredictable sensory features’), prediction error is calculated for each element of an event, determining which sensory details are stored.

### Modelling semantic memory

Existing semantic memory survives when the hippocampus is lesioned^43–45^, and hippocampal amnesics can describe remote memories more successfully than recent ones^8,84^, even if they might not recall them ‘episodically’^11^. This temporal gradient indicates that the semantic component of memories becomes HF-independent. In the model, EC lesions impair all truly episodic recollection since the return projections from the HF are required for the generation of sensory experiences. Here we describe how remote memories could be retrieved ‘in semantic form’ despite lesions including the hippocampus and the EC.

**Fig. 3:**
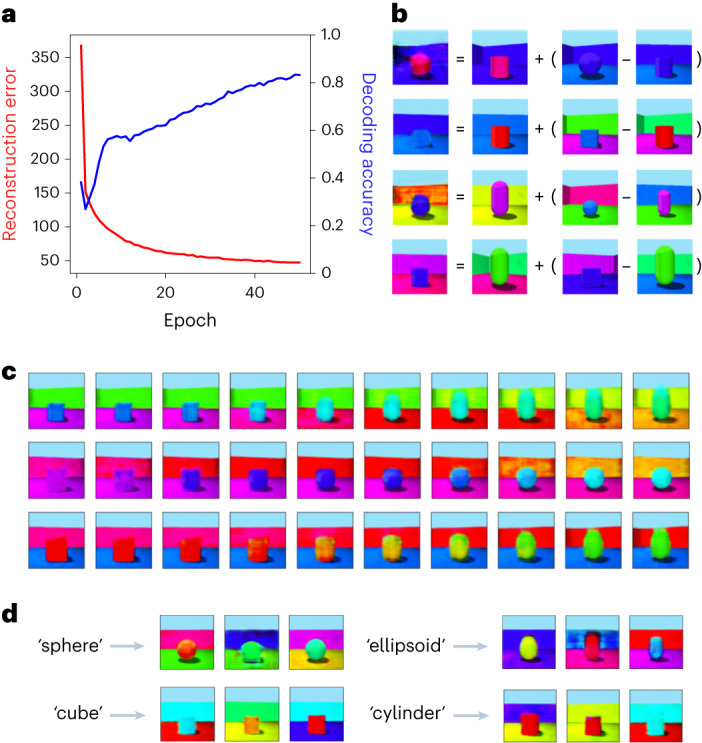
Learning, relational inference and imagination in the generative model. **a**, Reconstruction error (red) and decoding accuracy (blue) improve during training of the generative model. Decoding accuracy refers to the performance of a support vector classifier trained to output the central object’s shape from the latent variables, using 200 examples at the end of each epoch of generative model training. An epoch is one presentation of the training set of 10,000 samples from the hippocampus. **b**, Relational inference as vector arithmetic in the latent space. The three items on the right of each equation are items from the training data. Their latent variable representations are combined as vectors according to the equation, giving the latent variable representation from which the first item is generated. The pair in brackets describes a relation which is applied to the second item to produce the first. In the top row, the object shape changes from a cylinder to a sphere. In the second, the object shape changes from a cylinder to a cube, and the object colour from red to blue. In the third and fourth, more complex transitions change the object colour and shape, wall colour and angle. **c**, Imagining new items via interpolation in latent space. Each row shows points along a line in the latent space between two items from the training data, decoded into images by the generative network’s decoder. **d**, Imagining new items from a category. Samples from each of the shape categories of the support vector classifier in **a** are shown.

The latent variable representation of an event in the generative network encodes the key facts about the event and can drive semantic memory directly without decoding the representation back into a sensory experience (Fig. 1g). The output route via HF is necessary for turning latent variable representations in mPFC or alTL into a sensory experience, but the latent variables themselves could support semantic retrieval. Thus, when the HF (including the EC) is removed, the model can still support retrieval of semantic information (see section ‘Modelling brain damage’ for details). To show this, we trained models to predict attributes of each image from its latent vector. Figure 3a shows that semantic ‘decoding accuracy’ increases as training progresses, reflecting the learning of semantic structure as a by-product of learning to reconstruct the sensory input patterns (*r*_s_(48) = 0.997, *P* < 0.001, 95% confidence interval (CI) = 0.987, 1.000). While semantic memory is much more complex than simple classification, richer ‘semantic’ outputs such as verbal descriptions can also be decoded from latent variable representations of images^85,86^.

### Imagination, episodic future thinking and relational inference

Here we model the generation of events that have not been experienced from the generative network’s latent variables. Events can be generated either by external specification of latent variables (imagination) or by transforming the latent variable representations of specific events (relational inference). The former is simulated by sampling from categories in the latent space then decoding the results (Fig. 3d). The latter is simulated by interpolating between the latent representations of events (Fig. 3c) or by doing vector arithmetic in the latent space (Fig. 3b). This illustrates that the model has learnt some conceptual structure to the data, supporting reasoning tasks of the form ‘what is to *A* as *B* is to *C*?’, and provides a model for the flexible recombination of memories thought to underlie episodic future thinking^24^.

### Modelling schema-based distortions

**Fig. 4:**
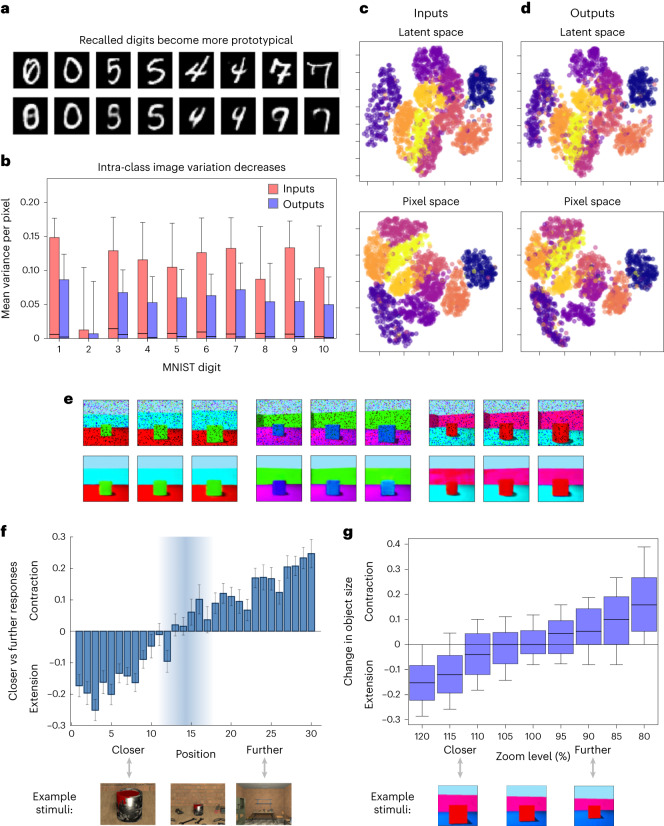
Generative network shows schema-based distortions. **a**, MNIST digits (top) and the VAE’s output for each (bottom). Recalled pairs from the same class become more similar. A total of 10,000 items from the MNIST dataset were encoded in the MHN, and 10,000 replayed samples were used to train the VAE. **b**, The variation within each MNIST class is smaller for the recalled items than for the original inputs. For each of the 10 classes, the variance per pixel is calculated across 500 images, and the 784 pixel variances are then plotted for each class before and after recall. In each boxplot, the box gives the interquartile range, its central line gives the median, and its whiskers extend to the 10th and 90th percentiles of the data. **c**,**d**, The pixel spaces of MNIST digits (bottom row) and the latent space of their encodings (top row) show more compact clusters for the generative network’s outputs (**d**) than for its inputs (**c**). Pixel and latent spaces are shown projected into 2D with UMAP^146^ and colour-coded by class. **e**, Examples of boundary extension and contraction. Top row: the noisy input images (from a held-out test set), with an atypically ‘zoomed out’ or ‘zoomed in’ view (by 80% and 120% on the left and right, respectively) for three original images. Bottom row: the predicted images for each input image, which are distorted towards the ‘typical view’ in each case. **f**, Adapted figure from ref. ^92^, showing the distribution of boundary extension vs contraction as a function of the viewpoint of an image. Specifically, the values are the average of ‘closer’ vs ‘further’ judgements, assigned −1 and 1, respectively, of an identical stimulus image in comparison with the remembered image (with 900 trials per position). Error bars give the standard error of the mean. Example stimuli are shown at the bottom. **g**, In our model, the VAE increases the estimated size of the central object in atypically ‘zoomed out’ views compared with the training data, and decreases it in atypically ‘zoomed in’ views, as in ref. ^92^. Two hundred images are used at each ‘zoom level’. See **b** for a description of boxplot elements.

The schema-based distortions observed in human episodic memory increase over time^6^ and with sleep^52^, suggesting an association with consolidation. Recall by the generative network distorts memories towards prototypical representations. Figure 4a–d shows that handwritten digits from the MNIST dataset^87^ ‘recalled’ by a VAE become more prototypical (MNIST is used for this because each image has a single category). Recalled pairs from the same class become more similar, that is, intra-class variation decreases (paired samples *t*-test *t*(7,839) = 60.523, *P* < 0.001, Cohen’s *d* = −0.684, 95% CI = 0.021, 0.022). The pixel space of MNIST digits before and after recall and the latent space of their encodings also show this effect. In summary, recall with a generative network distorts stimuli towards more prototypical representations even when no class information is given during training. As reliance on the generative model increases, so does the level of distortion.

Boundary extension and contraction exemplify this phenomenon. Boundary extension is the tendency to remember a wider field of view than was observed^88^, while boundary contraction is the opposite^89^. Unusually close-up views appear to cause boundary extension, and unusually far away ones boundary contraction^89^, although this is debated^90,91^. We modelled this by giving the generative network a range of new scenes that were artificially ‘zoomed in’ or ‘zoomed out’ compared with those in its training set; its reconstructions are distorted towards the ‘typical view’ (Fig. 4e), as in human data. Figure 4g shows the change in the object size in memory quantitatively, mirroring the findings in ref. ^92^ (Fig. 4f). (Note that the measure of boundary extension vs contraction used by ref. ^92^ is produced by averaging ‘closer’ vs ‘further’ judgements of an identical stimulus image in comparison with the remembered image, rather than the drawing-based measure we use, but the two measures are significantly correlated^89^.)

### Combining conceptual and unpredictable sensory features

In the extended model, memories stored in the hippocampal autoassociative network combine conceptual features (derived from the generative network’s latent variables) and unpredictable sensory features (those with a high reconstruction error during encoding) (Fig. 2). In these simulations, the conceptual features are simply a one-to-one copy of latent variable representations. (Since latent variable representations are not stable as the generative network learns, concepts derived from latent variables seem more likely to be stored than the latent variables themselves, so this is a simplification; see section ‘Extended model’ for further details.)

**Fig. 5:**
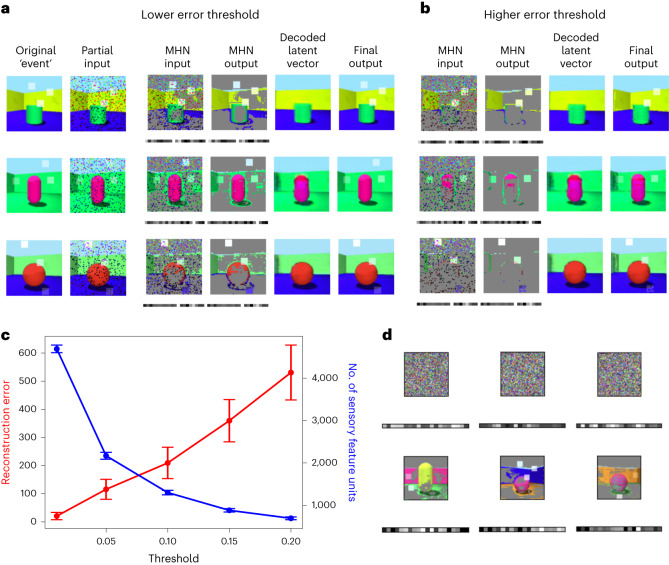
Retrieval dependence on reconstruction error threshold and replay in the extended model. **a**, The stages of recall are shown from left to right (see Fig. 2d), where each row represents an example scene. Each scene consists of a standard Shapes3D image with the addition of novel features (several white squares overlaid on the image with varying opacity). **b**, Repeating this process with a higher error threshold for encoding (with the same events and partial inputs) means fewer poorly predicted sensory features are stored in the autoassociative MHN, leading to more prototypical recall with increased reconstruction error. **c**, Average reconstruction error and number of sensory features (that is, pixels) stored in the autoassociative MHN against the error threshold for encoding. One hundred images are tested and error bars give the s.e.m. **d**, Replay in the extended model. The autoassociative network retrieves memories when random noise is given as input, as shown for three example inputs (upper row). As above, the square images show the poorly predicted sensory features and the rectangles below these display the latent variable representations (lower row).

Figure 5a,b shows the stages of recall in the extended model after encoding with a lower or higher prediction error threshold. After decomposing the input into its predictable (conceptual) and unpredictable (sensory) features, the autoassociative network performs pattern completion on the combined representation. The prototypical (that is, predicted) image corresponding to the retrieved conceptual features must then be obtained by decoding the associated latent variable representation into an experience via the return projections to the sensory neocortex. Next, the predictable and unpredictable elements are recombined, simply by overwriting the prototypical prediction with any unpredictable elements, via the connections from the sensory features to the sensory neocortex. The extended model is therefore able to exploit the generative network to reconstruct the predictable aspects of the event from its latent variables, storing only those sensory details that were poorly predicted in the autoassociative network. Equally, as the generative network improves, sensory features stored in the hippocampus may no longer differ significantly from the initial schematic reconstruction in the sensory neocortex, signalling that the hippocampal representation is no longer needed.

### Schema-based distortions in the extended model

The schema-based distortions shown in the basic model result from the generative network and increase with dependence on it, but memory distortions can also have a rapid onset^93,94^. In the extended model, even immediate recall involves a combination of conceptual and sensory features, and the presence of conceptual features induces distortions before consolidation of that specific memory.

In general, recall is biased towards the ‘mean’ of the class soon after encoding due to the influence of the conceptual representations (Fig. 5a,b). This is more pronounced when the error threshold for encoding is high, as there is more reliance on the ‘prototypical’ representations, resulting in the recall of fewer novel features. At a lower error threshold, more sensory detail is encoded, that is, the dimension of the memory trace is higher (*r*_s_(3) = −1, *P* < 0.001). This results in a lower reconstruction error (*r*_s_(3) = 1, *P* < 0.001), indicating lower distortion but at the expense of efficiency.

**Fig. 6:**
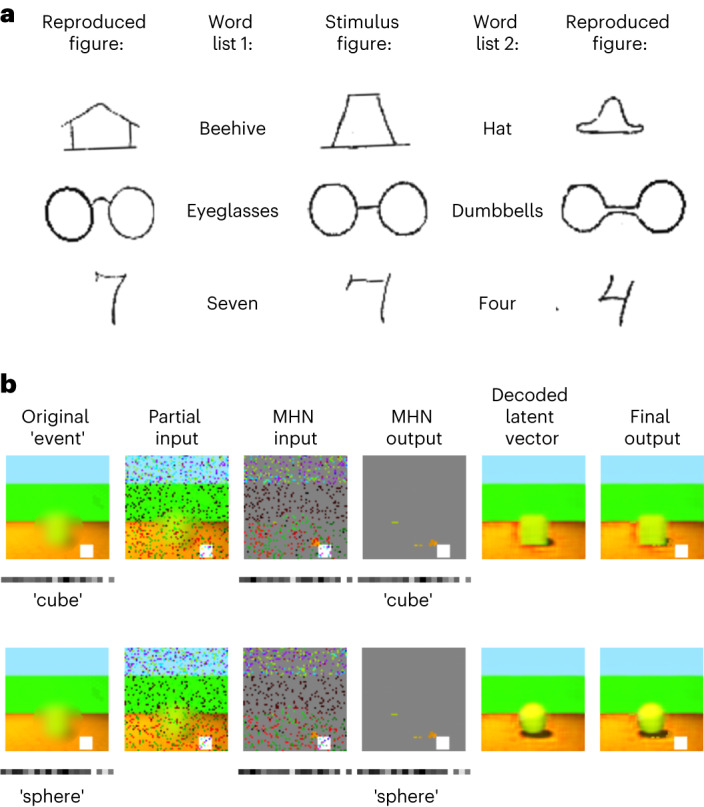
Schema-based distortions: effects of conceptual context in the extended model. **a**, Adapted figure from ref. ^95^ showing that recall of an ambiguous item (stimulus figure, centre) depends on its context at encoding (word from list 1, left; or list 2, right), as shown by drawing from memory (reproduced figure, far left and far right). **b**, Memory distortions in the extended model, when the original scene (containing an ambiguous blurred shape) is encoded with a given concept (cube, top; sphere, bottom), represented by the latent variables for that class. Then, a partial input is processed by the generative network to produce predicted conceptual features and the sensory features not predicted by the prototype for that concept (in this case, a white square) for input to the autoassociative MHN. However, pattern completion in the MHN reproduces the originally encoded sensory and conceptual features (cube, top; sphere, bottom), and these are recombined to produce the final output, which is distorted towards the encoded conceptual context.

External context further distorts memory. Reference ^95^ asked participants to reproduce ambiguous sketches. A context was established by telling the participants that they would see images from a certain category. After a delay, drawings from memory were distorted to look more like members of the context category. Figure 6b shows the result of encoding the same ambiguous image with two different externally provided concepts (a cube in the top row, a sphere in the bottom row), represented by the latent variables for each concept, as opposed to the latent variables predicted by the image itself as in Fig. 5a,b. During recall, the encoded concept is retrieved in the autoassociative network, determining the prototypical scene reconstructed by the generative network. This biases recall towards the class provided as context, mirroring Fig. 6a.

**Fig. 7:**
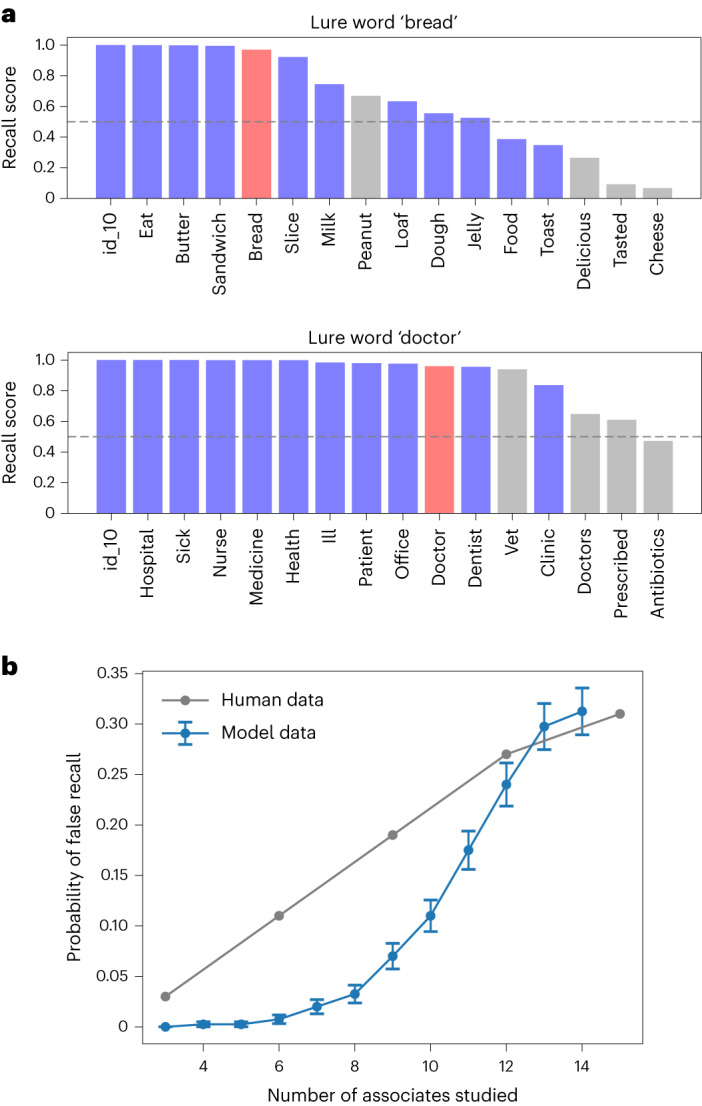
Modelling the DRM task. **a**, First, the VAE is trained to reconstruct simple stories^96^ converted to vectors of word counts, representing background knowledge. The system then encodes the lists as the combination of an ‘id_n’ term capturing unique spatiotemporal context, and the VAE’s latent variable representation of the word list. In each plot, recalled stimuli when the system is presented with ‘id_n’ are shown, with output scores treated as probabilities so that words with a score >0.5 (above dashed lines) are recalled. Words from the stimulus list are shown in blue, and lures in red. See Fig. 1 of Supplementary Information for results for the remaining DRM lists. **b**, The chance of recalling the lure word is higher when longer lists are encoded (blue). Each measurement is averaged across 400 trials (20 random subsets of each of the 20 DRM lists), and error bars give the s.e.m. This qualitatively resembles human data from ref. ^97^ (grey).

We also simulate the Deese–Roediger–McDermott (DRM) task^93,94^ in the extended model to demonstrate its applicability to non-image stimuli. In the DRM task, participants are shown lists of words that are semantically related to ‘lure words’ not present in the list; there is a robust finding that false recognition and recall of the lure words occur^93,94^. In the extended model, gist-based semantic intrusions arise as a consequence of learning the co-occurrence statistics of words. First, the VAE is trained to reconstruct the sets of words in simple stories^96^ converted to vectors of word counts, representing background knowledge. The system then encodes the experimental lists as the combination of an ‘id_n’ term capturing unique spatiotemporal context, and the VAE’s latent representation of each word list (respectively analogous to the stimulus-unique pixels and the VAE’s latent representation of each image in Fig. 5). As in the human data, lure words are often but not always recalled when the system is presented with ‘id_n’ (Fig. 7a), since the latent variable representations that generate the words in the list also tend to generate the lure word. The system also forgets some words and produces additional semantic intrusions. In addition, the chance of recalling the lure word is higher for longer lists, as in human data from ref. ^97^, as more related words provide a stronger ‘prior’ for the lure (Fig. 7b) (*r*_s_(10) = 0.998, *P* < 0.001, 95% CI = 0.982, 1.000).

### Modelling brain damage

Recent episodic memory is impaired following damage to the HF, whereas semantic memory, including the semantic content of remote episodes, appears relatively spared. In the model, the semantic form of a consolidated memory survives damage to the HF due to latent variable representations in the mPFC or the alTL (even if those in the EC are lesioned); Fig. 3a demonstrates how semantic recall performance improves with the age of a memory, reflecting the temporal gradient of retrograde amnesia (see section ‘Modelling semantic memory’). However, these semantic ‘facts’ cannot be used to generate an experience ‘episodically’ without the generative network’s decoder, in agreement with multiple trace theory^11^.

The extent of retrograde amnesia can vary greatly depending in part on which regions of the HF are damaged^98,99^. The dissociation of retrograde and anterograde amnesia in some cases suggests that the circuits for encoding memories and the circuits for recalling them via the HF only overlap partially^99^. For example, if the autoassociative network is damaged but not the generative network’s decoder, the generative network can still perform reconstruction of fully consolidated memories. This could explain varying reports of the gradient of retrograde amnesia when assessing episodic recollection (as opposed to semantic memory), if the generative network’s decoder is intact in patients showing spared episodic recollection of early memories^45^. Note that the location of damage within the generative network’s decoder also affects the resulting deficit in our model. In particular, patients with damage restricted to the hippocampus proper can (re)construct simple scenes but not more complex ones^23^.

Our model also shows the characteristic anterograde amnesia after hippocampal damage, as the hippocampus is required to initially bind features together and support off-line training of the generative model. Anterograde semantic learning would also be impaired by hippocampal damage (as the generative network is trained by hippocampal replay). While hippocampal replay need not be the only mechanism for schema acquisition, it would probably be much slower without the benefit of replay. However, semantic learning over short timescales may be relatively unimpaired, as it is less dependent on extracting regularities from long-term memory^100^.

In semantic dementia, semantic memory is impaired, and remote episodic memory is impaired more than recent episodic memory^101^. This would be consistent with lesions to the generative network, as recent memories can rely more on the hippocampal autoassociative network. However, the exact effects would depend on the distribution of damage across the various potential generative networks in the EC, mPFC and alTL. Of these, the alTL network is associated with semantic dementia, and the posterior medial network (corresponding to the generative network between the sensory areas and the EC) with Alzheimer’s disease^83^.

Finally, neuropsychological evidence suggests a distinction between familiarity and recollection, and furthermore a partial dissociation between different tests of familiarity; patients with selective hippocampal damage can exhibit recognition memory deficits in a simple ‘yes/no’ task with similar foils, but not in a ‘forced choice’ variant involving choosing the more familiar stimulus from a set^102^. This is consistent with the idea that lower prediction error in the neocortical generative network indicates familiarity, but retrieval of unique details from the hippocampus is required for more definitive recognition memory.

## Discussion

We have proposed a model of systems consolidation as the training of a generative neural network, which learns to support episodic memory, and also imagination, semantic memory and inference. This occurs through teacher–student learning. The hippocampal ‘teacher’ rapidly encodes an event, which may combine unpredictable sensory elements (with connections to and from the sensory cortex) and predictable conceptual elements (with connections to and from latent variable representations in the generative network). After exposure to replayed representations from the ‘teacher’, the generative ‘student’ network supports reconstruction of events by forming a schematic representation in the sensory neocortex from latent variables via the HF, with unpredictable sensory elements added from the hippocampus.

In contrast to the relatively veridical initial encoding, the generative model learns to capture the probability distributions underlying experiences, or ‘schemas’. This enables not just efficient recall, reconstructing memories without the need to store them individually, but also imagination (by sampling from the latent variable distributions) and inference (by using the learned statistics of experience to predict the values of unseen variables). In addition, semantic memory (that is, factual knowledge) develops as a by-product of learning to predict sensory experience. As the generative model becomes more accurate, the need to store and retrieve unpredicted details in the hippocampus decreases (producing a gradient of retrograde amnesia in cases of hippocampal damage). However, the generative network necessarily introduces distortion compared to the initial memory system. Multiple generative networks can be trained in parallel, and we expect this to include networks with latent variables in the EC, mPFC and alTL.

We now compare the model’s performance to the list of key findings from the introduction:

1. Gradual consolidation follows one-shot encoding: A memory is encoded in the hippocampal ‘teacher’ network after a single exposure, and transferred to the generative ‘student’ network after being replayed repeatedly (Fig. 1c,d).
2. Semantic memory becomes hippocampus-independent: The latent variable representations learned by the generative networks constitute the ‘key facts’ of an episode, supporting semantic memory (Fig. 3a).
3. Episodic memory remains hippocampus-dependent: Return projections to the sensory neocortex via the HF are required to decode the latent variable representations into a sensory experience (Fig. 1). (EC is required for even simple (re)construction, while the hippocampus proper helps to generate complex conceptually coherent scenes and retrieves unpredictable details that are not yet consolidated into the generative network; see section ‘Neural substrates of the model’.)
4. Shared substrate for episode generation: Generative models are a common mechanism for episode generation. Familiar scenes can be reconstructed and new ones can be generated by sampling or transforming existing latent variable representations (Fig. 3b–d), providing a model for imagination, scene construction and episodic future thinking.
5. Consolidation promotes inference and generalization: Relational inference corresponds to vector arithmetic applied to the generative network’s latent variables (Fig. 3b).
6. Episodic memories are distorted: We show how memory distortions arise from the generative network (Figs. 4, 6 and 7). This extends the model of ref. ^32^ to relate memory distortion to consolidation.
7. Association cortex encodes latent structure: Latent variable representations in the EC, mPFC, and alTL provide schemas for episodic recollection and imagination (via HF) and for semantic retrieval and inference.
8. Prediction error affects memory processing: The generative network is constantly calculating the reconstruction error of experiences^58,59^. Events that are consistent with the existing generative model require less encoding in the autoassociative hippocampal network (Fig. 5).
9. Episodic memories include conceptual features: When an experience combines a mixture of familiar and unfamiliar elements, both concepts and poorly predicted sensory elements are stored in the hippocampus via association to a specific memory unit.

Our model can be seen as an update to the complementary learning systems (CLS)^4^ framework to better account for points 3 to 9 above, reconciling the development of semantic representations in the neocortex (as in CLS) with the continued dependence on the hippocampal formation for episodic recall (as in multiple trace theory^11^). Furthermore, it provides a unified view of: (1) episode generation, (2) how episodic memories change over time and exhibit distortions and (3) how semantic and episodic information are combined in memory. We build on previous work exploring the role of generative networks in consolidation^18,19^, as models of the hippocampal formation^31–33^, as priors for episodic memory^35^ and as models of spatial cognition^22^.

A key aspect of the model is that multiple generative networks can be trained concurrently from a single autoassociative network (Fig. 2a) and may be optimized for different tasks. Thus, the latent representations in the mPFC and the alTL may be more closely linked to value or language than those in the EC^103,104^. These differences may arise from differences in network structure (for example, the degree of compression) or from additional training objectives that shape their representations^105^ (for example, the generative network with latent variables in the mPFC might be trained to predict task-relevant value in addition to the EC representations). We expect the generative networks to overlap closer to their sensory inputs/outputs, where general-purpose features are more useful, and diverge as the representations become more abstract (or task-specific if there are additional training objectives)^106^. This may involve a primary VAE with latent variables in the EC, with additional pathways from the higher sensory cortex to the EC routed via latent variables in the mPFC or the alTL.

Our model raises some fundamental questions: Does true episodic memory require event-unique detail, and does this require the hippocampus? Or can prototypical predictions qualify as memory rather than imagination? In the model, event-unique details are initially provided by the hippocampus but can also be provided by the generative network. For example, if you know that someone attended your 8th birthday party and gave you a particular gift, these personal semantic facts need not be hippocampal-dependent but could generate a scene with the right event-specific details, which would seem like episodic memory. The increasingly sophisticated generation of images from text using generative models^107^ suggests that episode construction from semantic facts is computationally plausible.

Episodic memories are defined by their unique spatiotemporal context^1^. In the model, spatial and temporal context correspond to conceptual features captured by place^108,109^ or time^110,111^ cells in the hippocampus and might be linked to latent variable representations formed in the EC, such as grid cells in the medial EC, which form an efficient basis for locations in real^31,112,113^ or cognitive spaces^31,54^, or temporal context representations in the lateral EC^114,115^. Events with specific spatial and temporal context can be generated from these latent variable representations, as has been modelled in detail for space^20–22^.

More generally, this work builds on the spatial cognition literature, in which place and head direction cells act as latent variables in a generative model^20–22^, allowing the generation of a scene from a specific viewpoint. References ^20–22^ explore how egocentric sensory representations could be transformed into allocentric latent variables before storage in the medial temporal lobe and conversely, how egocentric representations could be reconstructed from allocentric ones to support imagery. The latent representations learned through consolidation in our model correspond loosely to the allocentric representations, and the sensory representations produced by HF to the egocentric ones; only egocentric and sensory representations are directly experienced, whereas allocentric and semantic representations are useful abstractions that can also be exploited for efficient hippocampal encoding.

Our model simplifies the true nature of mnemonic processing in several ways. First, the interaction of sensory and conceptual features in the hippocampus and latent variables in the EC during retrieval could be more complex, with each type of representation contributing to pattern completion of the other as in interactions between items and contextual representations in the Temporal Context Model^116^, and might iterate over retrievals from both hippocampal and generative networks^50^. Second, our model distinguishes between ‘sensory’ and ‘conceptual’ representations in the hippocampus, respectively linked to the sensory neocortex at the input/output of the generative network and to the latent variable layer in the middle. In reality, a gradient of levels of representation in the hippocampus is more likely, from detailed sensory representations to coarse-grained conceptual ones, respectively linked to lower or higher neocortical areas^117^, and might map onto the observed functional gradients along the longitudinal axis of the hippocampus^118^. Third, our generative network uses back-propagation of the prediction error between output and input patterns to learn. Generative networks with more plausible (if less efficient) learning rules exist^67–69^, which have the advantage of producing a prediction error signal at each layer (between top–down prediction and bottom–up recognition), potentially allowing learning of concepts and exceptions at all levels of description. Fourth, considering consolidation as a continual lifelong process rather than during encoding of a single dataset introduces new complexities; these include the instability of latent representations and the prevention of catastrophic forgetting of already consolidated memories as new memories are assimilated into the generative network. The model could be extended to address this, for example, by using replay from the generative network as well as from the hippocampal network, which could reduce catastrophic forgetting and stabilize latent variable representations in both networks^33,119,120^, building on previous research on sleep and learning^121^. Fifth, we model semantic memory as prediction of categorical information for an ‘event’, but future work should model more complex semantic knowledge, for example, by decoding language from latent representations of multimodal stimuli^85,86^. In particular, the relationship between semantic memory for specific ‘events’ and the broader ‘web’ of general knowledge should be considered.

Episodic memories contain important sequential structure not modelled by our encoding and reconstruction of simple scenes. Future work could expand the model’s scope to sequential information as follows. A range of stimuli could be represented as sequences of arbitrary symbols (including language, spatial trajectories and transitions on a graph). A heteroassociative variant of an MHN, which is better suited to sequential data, could be used to store such stimuli. Specifically, the interpretation of an MHN that we use^64^ can capture sequential information if the projections from feature units to memory units correspond to the current state, but the projections from memory units back to feature units correspond to the next state so that one state retrieves the next^122–124^. With certain modifications based on previous work involving the role of temporal context in memory^116,125^, asymmetric MHNs can store sequences with complex repetitions and temporal correlations, such as language. We could then implement the student model as a sequential generative network trained to predict the next input during sequential replay (for example, GPT-2 (ref. ^126^)). Such networks capture relational structure, developing grid-like latent representations in spatial tasks^31^, and learn the gist of narratives. The sequential model could also be applied to phenomena such as event segmentation^127^ and memory distortions in narratives^6^. (Note that for more complex sequential data such as videos, pattern completion of both the current stimulus and the next stimulus would be required, potentially needing a combination of autoassociative and heteroassociative connectivity in the hippocampal network.)

Our model makes testable predictions. First, if participants learn stimuli generated from known latent variables, it predicts that these specific latent variable representations should develop in the association cortex over time (and that this representation would support, for example, vector arithmetic and interpolation). This could be tested by representational similarity analysis, which should reveal a more conceptual similarity structure developing in the association cortex through consolidation, as opposed to a similarity structure reflecting the sensory stimuli in earlier sensory cortices. If the stimuli also contained slight variation, that is, they were not entirely described by the latent variables, the development of a latent variable representation should be correlated with gist-based distortions in memory and anti-correlated with hippocampal processing of unpredictable elements.

Second, the model makes multiple predictions about the effects of brain damage. Just as boundary extension is reduced in patients with damage to the HF^128^ or the vmPFC^129^, we predict that other biases towards the ‘canonical view’ would be attenuated in such patients; for example, healthy controls would distort images with an atypical viewing angle towards a more typical angle in memory, but this would be reduced in, for example, hippocampal patients. Similarly, ambiguous images such as the duck/rabbit drawing ‘flip’ between interpretations in perception but are stable when held in imagery^72^, presumably due to maintained hippocampal conceptual representations. We predict that this conceptual stability in imagery would also be reduced in such patients. This could also extend to non-scene stimuli: if the ref. ^95^ task were tested with both healthy controls and patients with damage to the generative decoder, we would predict reduced contextual distortion in the latter. Furthermore, patients with an inaccurate generative model, for example, due to semantic dementia, might rely more on sensory features to compensate. (Note that the pattern of deficits would depend on both the nature of the priors encoded in the generative network and the error threshold for encoding. In some cases, damage to the generative network could produce atypical ‘priors’ rather than suppressing them. Thus, if the generative network is inaccurate but the error threshold for encoding is high, atypical distortions will be observed rather than a reduction in conceptual distortions.)

Third, the model suggests that the error threshold for encoding could vary depending on the importance of the stimuli or the amount of attentional resource available. For example, emotional salience could lower this threshold, with traumatic memories being encoded in greater sensory detail and with less contextual coherence^130,131^. Equally, conditions such as autism spectrum disorder, which are potentially attributable to hypo-priors^132^, might be associated with a lower prediction error threshold for veridical storage (and thus reduced conceptual influence on memory and increased sensory detail). In addition, reality monitoring deficits would change the perceived prediction error relative to reality, leading to atypical memory storage (for example, a reduced ability to compensate for prediction errors by storing sensory details).

Fourth, biological intelligence excels at generalizing from only a small number of examples. The model predicts that learning to generalize rapidly benefits from having a generative model that can create new examples, for example, by inferring variants (as in Fig. 3b) (see also ref. ^133^). Finally, the model suggests a link between latent spaces and cognitive maps^134^. For example, one might predict that the position of a memory in latent space is reflected in place and grid cell firing, as observed for other conceptual representations^54,134,135^.

In summary, our proposed model takes inspiration from recent advances in machine learning to capture many of the intriguing phenomena associated with episodic memory, its (re)constructive nature, its relationship to schemas, and consolidation, as well as aspects of imagination, inference and semantic memory.

## Methods

### Data

In the simulations, images represent events (except for the DRM^93,94^ task stimuli). The Shapes3D dataset^136^ was used throughout, except for the use of MNIST^87^ to explore certain distortions. Note that one MHN was used per dataset, and one generative model was trained per dataset from the corresponding MHN’s outputs.

### Basic model

In our model, the hippocampus rapidly encodes an event, modelled as one-shot memorization in an autoassociative network (an MHN). Then, generative networks are trained on replayed representations from the autoassociative network, learning to reconstruct memories by capturing the statistical structure of experienced events.

The generative networks used are variational autoencoders, a type of autoencoder with special properties such that randomly sampling values for the latent variables in the model’s ‘bottleneck’ layer generates valid stimuli^65^. Figure 3 of Supplementary Information, adapted from ref. ^137^, shows how directions in the latent space can correspond to meaningful transformations. While most diagrams show the VAE’s input and output layers in the sensory neocortex as separated (in line with conventions for visualizing neural networks), it is important to note that the input and output layers are in fact the same, as shown in Fig. 1b. There may be considerable overlap between the encoder and decoder, especially closer to the sensory neocortex, but we did not model this explicitly. The autoassociative model is an MHN, with the property that even random input values will retrieve one of the stored patterns via pattern completion. Specifically, we considered the biological interpretation of the MHN as feature units and memory units suggested by ref. ^64^ (see Supplementary Information for details).

We modelled consolidation as teacher–student learning, where the autoassociative network is the ‘teacher’ and the generative network is the ‘student’ trained on replayed representations from the ‘teacher’. We gave random noise (consisting of uniformly sampled values in each channel for each pixel) as an input to the MHN, then used the outputs of the network to train the VAE. (These outputs represent the high-level sensory representations activated by hippocampal pattern completion, via return projections to the sensory cortex.) The noise input to the autoassociative network could potentially represent random activation during sleep^138–140^. Attributes such as reward salience might also influence which memories are replayed but were not modelled here^141^.

During the encoding state in our simulations, images were stored in a continuous MHN with high inverse temperature, *β*, set to 20 (higher values of *β* produce attractor states corresponding to individual memories, while lower values of *β* make metastable states more likely). Reference ^63^ provides an excellent Python implementation of MHNs that we used in our code. During the ‘rest’ state, random noise was given as an input *N* times to the MHN, retrieving *N* attractor states from the network. (The distribution of retrieved attractor states was not tested but was approximately random, and very few spurious attractors were observed with sufficiently high inverse temperature.) In the main simulations, 10,000 items from the Shapes3D dataset were encoded in the MHN, and 10,000 replayed states were used to train the VAE (that is, *N* is 10,000). (Rather than replaying new samples from the MHN at each epoch of the VAE’s training, a single set of samples was used for efficiency and simplicity.)

A VAE was then trained on the ‘replayed’ images from the MHN, using the Keras API for TensorFlow^142^. The loss function (that is, the error minimized through training) is the sum of two terms, the reconstruction error and the Kullback–Leibler divergence^65^; the former encourages accurate reconstruction, while the latter (which measures the divergence between the latent variables and a Gaussian distribution) encourages a latent space one can sample from. Specifically, the reconstruction loss in our model is a mean absolute error loss. (Note that the terms reconstruction error and prediction error are used interchangeably throughout.)

The stochastic gradient descent method used was the AMSGrad variant of the Adam optimizer with early stopping enabled, for a maximum of 50 epochs (where an epoch is a complete pass through the training set). A latent variable vector length of 20, learning rate of 0.001 and Kullback–Leibler weighting of 1 were used in the main results. The variational autoencoders were not optimized for performance, as their purpose is illustrative (more data and hyperparameter tuning would be likely to improve reconstruction accuracy). Architectural choices within the VAE were not principled but were based on successful architectures for similar stimuli in the literature. See Supplementary Information for details of the VAE’s architecture. The VAEs were trained using gradient descent and back-propagation as usual; while this method is biologically implausible due to its non-local nature, more plausible learning algorithms might be feasible^143^.

While this was not modelled explicitly, once the generative network’s reconstruction error is sufficiently low, the hippocampal trace is unnecessary. As a result, it could be ‘marked for deletion’ or overwritten in some way, freeing up capacity for new encodings. However, we did not simulate decay, deletion or capacity constraints in the autoassociative memory part of the model. In these simulations, the main cause of forgetting would be interference from new memories in the generative model.

Note that throughout the simulations, the input to recall was a noisy version of the encoded stimulus image. Specifically, noise was added by replacing a random fraction (0.1 unless stated otherwise) of values in the image array with zero.

While we used only one modality at a time (imagery for the majority of simulations, text for the DRM task), our model is compatible with the multimodal nature of experience, as multimodal inputs to VAEs are possible, which result in a multimodal latent space^144^. This could reflect the multimodal nature of concept cells in the hippocampus^61^.

### Modelling semantic memory

We modelled semantic memory as the ability to decode latent variables into semantic information without the need to reconstruct the event episodically.

Decoding accuracy was measured by training a support vector machine to classify the central object’s shape from the network’s latent variables, using 200 examples at the end of each epoch and measuring classification accuracy on a held-out test set. (Notably, there was good performance with only a small amount of training data when decoding the latent variables, compared with decoding alternative representations such as the sensory input or intermediate layer activations, that is, few-shot learning is possible by making use of compressed ‘semantic’ representations. See Fig. 2 of Supplementary Information.)

### Modelling imagination and inference

In the generative network, new items can either be generated from externally specified (or randomly sampled) latent variables (imagination), or by transforming the latent variable representations of specific events (relational inference). The former was simulated by sampling from categories in the latent space, then decoding the results (Fig. 3d). The latter was simulated by interpolating between the latent representations of events (Fig. 3c) or by doing vector arithmetic in the latent space (Fig. 3b).

Examples of the four different object shapes were generated by Monte Carlo sampling for simplicity, that is, samples from the latent space were classified by the semantic decoding classifier, and examples that activate each category are displayed. (Note that there are many alternative ways to do this, for example, by extracting the decision boundaries from the classifier and sampling within the region corresponding to each class.) Generating imagined scenes from more naturalistic inputs, for example, natural language descriptions, would require a much more sophisticated text to the latent space model, but recent machine learning advances suggest that this is possible^145^.

To demonstrate interpolation, each row of Fig. 3c shows items generated from latent variables along a line in the latent space between two real items from the training data. To demonstrate vector arithmetic, each equation in Fig. 3b shows ‘result = vector_*A*_ + (vector_*B*_ − vector_*C*_)’ (reflecting relational inference problems of the form ‘what is to *A* as *B* is to *C*?’), where the result is produced by taking the relation between vector_*B*_ and vector_*C*_, applying that to vector_*A*_ and decoding the result. In other words, the three items on the right of each equation in Fig. 3b are real items from the training data. Their latent variable representations are combined as vectors according to the equation shown, giving the latent variable representation from which the first item is generated. Thus, the pair in brackets describes a relation that is applied to the first item on the right to produce the new item on the left of the equation.

### Modelling schema-based distortions

Items recalled by the generative network become more prototypical, a form of schema-based distortion. This can be shown simply in the basic model, using the MNIST digits dataset^87^ to exemplify ten clearly defined classes of items (Fig. 4). To show this quantitatively, we calculated the intra-class variation, measured as the mean variance per pixel, within each MNIST class before and after recall, for 5,000 images from the test set. As expected, the intra-class variation was smaller for the recalled items than for the original inputs. (See Supplementary Information for details of the model architecture.)

To visualize this, we projected the pixel and latent spaces before and after recall (of 2,000 images from the MNIST test set) into two dimensions (2D) with uniform manifold approximation and projection (UMAP)^146^, a dimensionality reduction method, and colour-coded them by class (Fig. 4c,d). The pixel space of MNIST digits (bottom row) and the latent space of their encodings (top row) showed more compact clusters for the generative network’s outputs (Fig. 4d) than for its inputs (Fig. 4c).

### Modelling boundary extension and contraction

Boundary extension is the tendency to remember a wider field of view than was observed for certain stimuli^88^, while boundary contraction is the tendency to remember a narrower one^89^. Whether boundaries are extended or contracted seems to depend on the perceived distance of the central object, with unusually close-up (that is, ‘object-oriented’) views causing boundary extension, and unusually far away (that is, ‘scene-oriented’) views causing boundary contraction^89^.

We tested boundary extension and contraction in the basic model by giving it a range of artificially ‘zoomed in’ or ‘zoomed out’ images, adapted from Shapes3D scenes not seen during training, and observing the outputs. The ‘zoomed in’ view was produced by removing *n* pixels from the margin. The ‘zoomed out’ view was produced by extrapolating the pixels at the margin outwards by *n* additional pixels. (In both cases, the new images were then resized to the standard size.) The zoom level is the ratio of the central object size in the output image to the size in the original image, given as a percentage; for example, an image with a zoom level of 80% or a ratio of 0.8 was produced by adding a margin so that the object size is 80% of the original size. As the Shapes3D images are of width and height 64, the number of pixels to add or remove was calculated as ‘margin = (32/ratio) − 32’.

In Fig. 4g, the change in object size between the noisy input and output was estimated as follows: first the image was converted to a few colours by *k*-means clustering of pixels. Then, the colour of the central object was determined by finding the predominant colour in a particular central region of the image. A 1D array of pixels corresponding to a vertical line at the horizontal midpoint of the image was processed to identify the fraction of pixels of the central object colour. This enabled us to calculate the change in object size, which we plotted against the degree of ‘zoom’. (For this object size estimation approach to work, we filtered the Shapes3D dataset to images where the object colour was different from both the wall and floor colour, and additionally to cubes to minimize shadow.)

Note that the measure of boundary extension vs contraction displayed in Fig. 4f, reproduced from ref. ^92^, was not based on the degree of distortion, but was produced by averaging ‘closer’ vs ‘further’ judgements of an identical stimulus image in comparison to the remembered image. This differs from our measure in Fig. 4g, which instead corresponds to the drawing-based measure in ref. ^89^; however, these measures have been shown to be correlated^89^.

Figure 4e shows a few examples of boundary extension and contraction. In the left- and right-hand images of each set, the margin *n* is chosen such that the central object is 80% and 120% of the original size, respectively.

### Extended model

The extended model was designed to capture the fact that memory traces in the hippocampus bind together a mixture of sensory and conceptual elements, with the latter encoded by concept cells^61^, and the fact that schemas shape the reconstruction of memories even before consolidation, as shown by the rapid onset of schema-based distortions^93,94^.

In the extended model, each scene was initially encoded as the combination of a predictable and an unpredictable component. The predictable component consisted of concepts captured by the latent variables of the generative network, and the unpredictable component consisted of parts of the stimuli that were poorly predicted by the generative network. Thus, the MHN model has both conceptual and sensory feature units, which store the predictable and unpredictable aspects of memory, respectively. While memories may eventually become fully dependent on the generative model, consolidation can be a prolonged process during which the generative network provides schemas for reconstruction and the autoassociative network supports new or detailed information not yet captured by schemas. (The VAE trained in the basic model simulations was used in the extended model simulations described below.)

How did encoding work in our simulations? For a new image, the prediction error of each pixel was calculated by the VAE (simply the magnitude of the difference between the VAE’s input and output). Those pixels with a reconstruction error above the threshold constituted the unpredictable component, while the VAE’s latent variables constituted the predictable component, and these components were combined into a single vector and encoded in the MHN. Note that when the threshold is zero, the reconstruction is guaranteed to be perfect, but as the threshold increases, the reconstruction decreases in accuracy.

How did recall work before full consolidation? After decomposing the input into its predictable (conceptual) and unpredictable (sensory) components, as described above, the autoassociative network could retrieve a memory. The image corresponding to the conceptual component was then obtained by decoding the stored latent variables. Next, the predictable and unpredictable elements were recombined, simply by overwriting the initial schematic reconstruction in the sensory neocortex with any stored (that is, non-zero) sensory features in the hippocampus. Figure 5a,b shows this process. The lower the error threshold for encoding sensory details, the more information was stored in the autoassociative network, reducing the reconstruction error of recall (see also section ‘Modelling schema-based distortions’).

How did replay work? When the autoassociative network was given random noise, both the unpredictable elements and the corresponding latent variables were retrieved. In Fig. 5d, the square images show the unpredictable elements of MNIST images and the rectangles below these display the vector of latent variables. (As the generative model improves, the presence of hippocampal sensory features that no longer differ from the initial reconstruction indicates that the hippocampal representation is no longer needed, but this was not simulated explicitly.)

We note that the latent variable representation is not stable as the generative network learns. If some latent variables are stored in the autoassociative network while the VAE continues to change, the quality of the VAE’s reconstruction will gradually worsen; this is also a feature of previous models^42^. Some degree of degradation may reflect forgetting, but consolidation can be a prolonged process and hippocampal representations can persist in this time. Therefore, we think that concepts derived from latent variables are more likely to be stored than the latent variables themselves, promoting the stability of hippocampal representations. (For example, in humans, language provides a set of relatively persistent concepts, stabilized by the need to communicate.) Projections from the latent variables can classify attributes with only a small amount of training data (see section ‘Modelling semantic memory’); we suggest that there could be a two-way mapping between latent variables and concepts, which supports categorization of incoming experience as well as semantic memory. However, for simplicity, the conceptual features were simply a one-to-one copy of latent variable representations in these simulations. It may also be possible to stabilize the latent variable representations by reducing catastrophic forgetting in the generative network, for example, by using generative as well as hippocampal replay^33,119,120^, with the generative network trained on its own self-generated representations in addition to new memories. This builds on previous research suggesting that certain stages of sleep are optimized to preserve remote memories, while others consolidate new ones^121^. This could reduce interference of new learning with remote memories in the generative network, as well as make hippocampal representations in the extended model more stable.

### Modelling schema-based distortions in the extended model

#### Carmichael experiment

We demonstrated the contextual modulation of memory (as in ref. ^95^) in the extended model by manipulating the conceptual component of an ‘event’. To model an external conceptual context being encoded, the original image was stored in the autoassociative network along with activation of a given concept (a cube or a sphere), represented as the latent variables for that class. While in most simulations the latent variables stored in the MHN were simply the output of the VAE’s encoder, here an external context activated the conceptual representation, consistent with activity in the EC, mPFC or alTL driven by extrinsic factors.

During recall, a noisy input was processed by the generative network to produce a predicted conceptual feature and the sensory features not predicted by the prototype for that concept, for input to the autoassociative MHN. Pattern completion in the MHN produced the originally encoded sensory and conceptual features, and these were recombined to produce the final output.

#### DRM experiment

The DRM task is a classic way to measure gist-based memory distortion^93,94^. Here we demonstrated the rapid onset of semantic intrusions in the extended model, coming about as a consequence of learning the co-occurrence statistics of words in a text dataset representing ‘background knowledge’. This followed on from previous work showing that VAEs produce semantic intrusions^32^.

In brief, the DRM task involved showing participants a list of words that were semantically related to a ‘lure word’, which was not present in the list. There was a tendency for both false recognition and false recall of the lure word. We focused on modelling the recall task, but the same model could be extended to recognition (with recognition memory measured by the reconstruction error of the network).

The generative network was pre-trained on a set of word lists extracted from simple stories^96^, representing learning from replayed memories before the DRM stimuli (although replay was not simulated explicitly). Words occurring in <0.05% or >10% of documents were discarded to keep the vocabulary to a manageable size of 4,206 words (this meant that some rarer words in the DRM lists were removed). The word lists were converted to vectors of word counts of length 4,206, in which the value at index *i* of the vector for a given list indicated the count of word *i* in the document. As these representations ignore word order, a sequential model was not required (however, this prevented exploring the effect of list position on recall).

Specifically, the variational autoencoder used for this simulation consisted of an input layer followed by a dropout layer^147^ projecting to 300 latent variables (sampled from representations of the mean and log variance vectors as usual), and then to an output layer with a sigmoid activation so that predictions were between 0 and 1, with L1 regularization to promote sparsity in this layer. As above, this was implemented using the Keras API for the TensorFlow library^142,148^, with the VAE trained to reconstruct input vectors in the usual way.

Following pre-training of the generative network, the system encoded the DRM stimuli, with each of the 20 word lists represented as vectors of word counts. One important detail was the addition of a term, given by ‘id_n’ for the *n*th document in the corpus, representing the unique spatiotemporal context of each word list. (Note that this is a highly simplified representation of the spatiotemporal context^116^ for illustration.) This enabled recall to be modelled by presenting the network with the ‘id_n’ term, and seeing which terms were retrieved.

In the extended model, the latent representation of the word list was encoded in the MHN as the conceptual component, while the unique ‘id_n’ terms were encoded veridically (as vectors of word counts of length 4,226—the original vocabulary size plus the 20 new ‘id_n’ terms—with 1 at ‘id_n’ and 0 elsewhere). The sparse vector representing the unexpected ‘id_n’ term is analogous to the sparse arrays of poorly predicted pixels in the main simulations of the extended model.

When the MHN was given ‘id_n’ as an input, it retrieved the hippocampal trace consisting of ‘id_n’ together with the latent representation of the word list. The latent representation was then decoded to produce the outputs shown in Fig. 7a (a dashed line shows the threshold for recall, interpreting the output as a probability so that words with an output >0.5 are recalled). As in the human data, lure words were often but not always recalled. The system also forgot some words and produced additional semantic intrusions, for example, ‘vet’ in the case of the ‘doctor’ list.

To test the effect of varying the number of associates, as in ref. ^97^, subsets of the DRM lists were encoded in the way described above. Specifically, to test the probability of lure recall with *n* associates studied, *n* items from each DRM list were encoded. For each list, this was repeated for 20 randomly sampled combinations of *n* items. Once again, recall was tested by giving the system ‘id_n’ as an input.

### Reporting summary

Further information on research design is available in the Nature Portfolio Reporting Summary linked to this article.

### Supplementary information

Supplementary Information Supplementary results (including two figures) and further model information (including one figure).

Reporting Summary

## Data Availability

The following datasets (all covered by the Creative Commons Attribution 4.0 License) were used in the simulations: MNIST^88^: https://www.tensorflow.org/datasets/catalog/mnist Shapes3D^137^: https://www.tensorflow.org/datasets/catalog/shapes3d ROCStories^97^: https://cs.rochester.edu/nlp/rocstories

## Code availability

Code for all simulations can be found at https://github.com/ellie-as/generative-memory. Some diagrams were created using BioRender.com.
