# Supplementary material for: A generative model of memory construction and consolidation
Source: Nat Hum Behav. 2024 Jan 19;8(3):526–43. doi: 10.1038/s41562-023-01799-z (PMC10963272; doi:10.1038/s41562-023-01799-z)
Supplement: Supplementary file 2 — Reporting Summary [file 41562_2023_1799_MOESM2_ESM.pdf]

nature portfolio

Corresponding author(s): Eleanor Spens

Last updated by author(s): 17/11/2023

Reporting Summary

Nature Portfolio wishes to improve the reproducibility of the work that we publish. This form provides structure for consistency and transparency in reporting. For further information on Nature Portfolio policies, see our Editorial Policies and the Editorial Policy Checklist.

Statistics

For all statistical analyses, confirm that the following items are present in the figure legend, table legend, main text, or Methods section.

n/a	Confirmed	
☐	☒	The exact sample size (*n*) for each experimental group/condition, given as a discrete number and unit of measurement
☒	☐	A statement on whether measurements were taken from distinct samples or whether the same sample was measured repeatedly
☐	☒	The statistical test(s) used AND whether they are one- or two-sided<br>*Only common tests should be described solely by name; describe more complex techniques in the Methods section.*
☒	☐	A description of all covariates tested
☒	☐	A description of any assumptions or corrections, such as tests of normality and adjustment for multiple comparisons
☐	☒	A full description of the statistical parameters including central tendency (e.g. means) or other basic estimates (e.g. regression coefficient) AND variation (e.g. standard deviation) or associated estimates of uncertainty (e.g. confidence intervals)
☐	☒	For null hypothesis testing, the test statistic (e.g. *F*, *t*, *r*) with confidence intervals, effect sizes, degrees of freedom and *P* value noted<br>*Give P values as exact values whenever suitable.*
☒	☐	For Bayesian analysis, information on the choice of priors and Markov chain Monte Carlo settings
☒	☐	For hierarchical and complex designs, identification of the appropriate level for tests and full reporting of outcomes
☐	☒	Estimates of effect sizes (e.g. Cohen's *d*, Pearson's *r*), indicating how they were calculated

Our web collection on statistics for biologists contains articles on many of the points above.

Software and code

Policy information about availability of computer code

Data collection	Code for data collection (i.e. the retrieval and pre-processing of datasets from online repositories) can be found at https://github.com/ellie-as/generative-memory.
Data analysis	Code for all simulations, as well as instructions to replicate the results, can be found at https://github.com/ellie-as/generative-memory. A list of all Python libraries used and their versions can be found at https://github.com/ellie-as/generative-memory/blob/main/requirements.txt. Diagrams were created using BioRender.com.

For manuscripts utilizing custom algorithms or software that are central to the research but not yet described in published literature, software must be made available to editors and reviewers. We strongly encourage code deposition in a community repository (e.g. GitHub). See the Nature Portfolio guidelines for submitting code & software for further information.

Data

Policy information about availability of data

All manuscripts must include a data availability statement. This statement should provide the following information, where applicable:

- Accession codes, unique identifiers, or web links for publicly available datasets
- A description of any restrictions on data availability
- For clinical datasets or third party data, please ensure that the statement adheres to our policy

The following datasets (all covered by the Creative Commons Attribution 4.0 License) were used in the simulations: 1) MNIST (LeCun et al., 2010) https://

www.tensorflow.org/datasets/catalog/mnist, 2) Shapes3D (Burgess & Kim, 2018) https://www.tensorflow.org/datasets/catalog/Shapes3D, and 3) ROC Stories (Mostafazadeh et al., 2016) https://cs.rochester.edu/nlp/rocstories.

Research involving human participants, their data, or biological material

Policy information about studies with human participants or human data. See also policy information about sex, gender (identity/presentation), and sexual orientation and race, ethnicity and racism.

Reporting on sex and gender	This is a computational study using only secondary data, with no modelling related to sex and/or gender.
Reporting on race, ethnicity, or other socially relevant groupings	This is a computational study using only secondary data, with no modelling related to race/ethnicity/other socially relevant groupings.
Population characteristics	The manuscript discusses how several neurological conditions relate to our model, but data relating to these conditions is not used directly.
Recruitment	Computational study using only secondary data - not applicable.
Ethics oversight	This study is in accordance with the ethics regulations at the Institute of Cognitive Neuroscience.

Note that full information on the approval of the study protocol must also be provided in the manuscript.

Field-specific reporting

Please select the one below that is the best fit for your research. If you are not sure, read the appropriate sections before making your selection.

☒ Life sciences ☐ Behavioural & social sciences ☐ Ecological, evolutionary & environmental sciences

For a reference copy of the document with all sections, see nature.com/documents/nr-reporting-summary-flat.pdf

Life sciences study design

All studies must disclose on these points even when the disclosure is negative.

Sample size	In this computational study, sample sizes sufficient to give statistically significant results were chosen.
Data exclusions	We did not exclude any outliers in our analysis. Where our analysis was restricted to a subset of a particular dataset, this is stated in the Methods.
Replication	The code provided on Github (see above) allows the findings to be replicated.
Randomization	Randomisation in the usual sense does not apply as this is a computational study using only secondary data. However in several cases we took repeated random samples from datasets to ensure our results were robust.
Blinding	Computational study using only secondary data - not applicable.

Reporting for specific materials, systems and methods

We require information from authors about some types of materials, experimental systems and methods used in many studies. Here, indicate whether each material, system or method listed is relevant to your study. If you are not sure if a list item applies to your research, read the appropriate section before selecting a response.

Materials & experimental systems

n/a	Involved in the study	
☒	☐	Antibodies
☒	☐	Eukaryotic cell lines
☒	☐	Palaeontology and archaeology
☒	☐	Animals and other organisms
☒	☐	Clinical data
☒	☐	Dual use research of concern
☒	☐	Plants

Methods

n/a	Involved in the study	
☒	☐	ChIP-seq
☒	☐	Flow cytometry
☒	☐	MRI-based neuroimaging
